# Hospital and Health News

**Published:** 1924-05

**Authors:** 


### HOSPITAL AND HEALTH NEWS.

### £15,000 for University College Hospital.

Mr. Geoffrey Duveen has offered £15,000 to University College Hospital, to provide the latest modern treatment.

### A Ward for Lascars.

Mr. P. J. Suighanee, of Poona, has given a lakh of rupees (£6,732) to build and endow a ward for lascar patients at the Tilbury Hospital.

### Two Gifts of £10,000.

Two gifts of £10,000 have been received in succession by the Chesterfield, North Derbyshire, Royal Hospital. Mr. Charles Markham gave the first, and the second came under the will of the late Mr. Thomas Murphy.

### New Secretary of Willesden Hospital.

Mr. A. Hearne, assistant secretary of St. Mary's Hospital, has been appointed secretary of the Willesden General Hospital. There were nearly two hundred applications for the post, which became vacant through the resignation of Mr. Edgell Brown.

### Territorial Army Nursing Service.

The 1924 Regulations for admission to the Territorial Army Nursing Service have recently been published through the War Office. Details are given of the necessary qualifications of candidates, the conditions of their appointment, their training, uniform and pay.

### M.P.'s Lay Foundation Stones of Hospital.

A minister, Mr. F. W. Jowett (First Commissioner of Works), two women M.P.'s (Lady Terrington and Miss Dorothy Jewson), and an M.P.'s wife (Lady Wise, wife of the Unionist Member for Ilford) laid the four foundation-stones of the new Maternity Hospital at Ilford.

### Why Not Women as Hospital Chairmen ?

Prince Arthur of Connaught, the new President of the Middlesex Hospital, speaking to the Governors, said that he regretted that women were not yet allowed to be chairmen of the great London hospitals. The Princess, who was a registered nurse and had had experience at several hospitals, would, he felt, fill the chairmanship better than he.

### Future Plans of Royal Free Hospital.

The future plans of the Royal Free Hospital include a department for middle-class patients at a reasonable charge on the site adjoining the hospital which has been given to it. The Marlborough Maternity Section now in Endsleigh Street will be moved to the main hospital when the tenancy expires. The Hospital's Medical School will celebrate its jubilee next October.

### Death of a Hospital Chairman.

By the death abroad of Mr. Carrington-Smith, the Acton Hospital has lost its founder, who remained its friend and contributed handsomely to its enlargement. He had been president of the hospital for 22 years. Lieut.-Col. Sword has been appointed, out of 360 applicants, secretary to the hospital. He is stated to be well acquainted with accounts and office routine, and to have shown ability as an organiser.

### Saving an Ancient Institution.

A last effort has been made to save the Farringdon General Dispensary and Lying-in Charity, though all the formalities for closing it were complete. The rector of St. Andrew's, Holborn, who presided at the annual meeting, made this announcement, which was received with relief, for the institution is nearly a hundred years old. Despite heavy debt and £1,000 needed to repair the building, the effort to continue the work has been successful.

### Westminster Hospital Appeal.

The appeal of the Governors of Westminster Hospital for £16,000, which will entitle them to an additional £16,000 generously offered by Sir H. M. Mallaby-Deeley, has met with a wonderful response from rich and poor in all parts of the country; but much of the required amount has still to be received by the Treasurer, Sir Robert Hudson, G.B.E., at Westminster Hospital, Broad Sanctuary, S.W. 1. At the hospital there are four new wards which have not yet been named, and the governors will be glad to hear from anyone who wishes to perpetuate a name by giving it to a hospital ward.

### Extension of Sunlight Treatment.

Dr. C. W. Saleeby, the well-known advocate of sunlight as a necessity for health and a remedy for many diseases, gave an address at Bournemouth in connection with the scheme for extending the work of the Royal Victoria and West Hants Hospital by building new wards for sunlight treatment. In his speech he declared that the scheme would inaugurate a new movement in the same direction throughout the country.

### Annual Conference on Tuberculosis.

The tenth Annual Conference of the National Association for the Prevention of Tuberculosis will be held in London on July 3 and 4. The main subject under consideration will be "The Part Played by Training Colonies in the Treatment of Tuberculosis," and the Conference will take the form of a practical demonstration of the work carried out at the Training Colony founded and maintained by the National Association at Burrow Hill, Frimley.

### For the Sick Room.

Sick-feeders and other articles made in "Bisto" earthenware for use in the sick room are manufactured by Messrs. Bishop and Stonier, Ltd., of Hanley. The "Bisto" and "Trent" sick-feeders are registered articles of a new style and improved form. They will not spill and are easily cleaned. Both are obtainable in two sizes, the former at 30s. and 36s., the latter at 36s. and 42s. The London showrooms of the firm are at Ely House, 13 Charterhouse Street, Holborn Circus, E.C. 1.

### Lectures by Sir Robert Armstrong-Jones.

Four lectures by Sir Robert Armstrong-Jones, M.D., will be held at Gresham College, Basinghall Street, E.C. 2, on April 29 and 30, and May 1 and 2, on "The Brain and the Mind." The first will be on "The Brain or the Physical Basis of the Mind"; the second, "The Mind"; the third, "Defects and Abnormal States of Mind"; and the fourth, "Spiritual Healing, Mental Healing, Physical Healing." The lectures begin at 6 p.m. each evening and are free to the public.

### International Health Conference at Wembley.

A conference to be held in connection with the British Empire Exhibition has been organised by the People's League of Health on "The Latest Knowledge regarding the Causes, Treatment and Prevention of Disease." The programme of the conference will occupy four days, leading up to and including Empire Day, May 24. It has been planned on international lines, and pioneers of famous methods of treatment will speak, together with well-known Empire representatives. Viscount Burnham, C.B.E., who is a vice-president of the League, will preside.

### New Hospital Wing.

The South London Hospital for Women and Children is fortunate in having generous friends who have given to it premises suitable for converting into a new wing and a sum of £12,000 to cover the cost of adapting the premises for hospital work. The maintenance of this new wing, which provides 36 beds for surgical patients and the necessary accommodation for additional nurses, will add no less than £6,000 to the yearly expenditure; a fund has been inaugurated, therefore, by which it is hoped to raise the required sum. The scheme is to enrol helpers who will collect a penny a week from householders throughout the year. By this means it is estimated that 1,000 helpers collecting 2s. weekly will provide a regular income of £5,000

### More X-Ray Heroes.

The Carnegie Hero Fund Trust has awarded a certificate and pension to Mr. Alfred Smith, a well-known Coventry radiologist, whose researches into the treatment of disease by X-ray culminated in his contracting X-ray dermatitis and the amputation of his right leg. The French Government has conferred the Cross of Chevalier of the Legion of Honour upon Emile Deligny, an X-ray worker at the Mustapha Hospital, Algiers. He has worked with the X-rays for nineteen years and, as the citation in the *Journal Officiel* mentions, gave "an incomparable example of courage and professional abnegation and devotion." Already he has lost his right arm, and is threatened with blindness, while other amputations will, it is feared, be necessary later on.

### The National League of Health.

We have received the Annual Report for 1923 of the National League of Health, Maternity and Child Welfare. Formed nineteen years ago, it has now a veritable army of officials and health visitors and a net-work of Infant Welfare Centres and Ante-natal Clinics with more than 350,000 mothers under their care. As a result, the report shows, there has been an astounding reduction in infant mortality, marked efforts to secure a clean milk supply for the country, and a quickening of public interest in all matters of public health.

### A District Nursing Directory.

Hospital almoners and others who desire to secure home nursing for cases of sickness in the poorer districts of London will be glad to know that the Directory, originally compiled and issued by the Central Council for District Nursing some years ago, has now been reissued with a supplement which brings up to date the information as to the appropriate Nursing Association for every street in London. Some 30,000 streets and places within the County of London are included in the Directory, which has an index giving the names and addresses of all the recognised District Nursing Associations and a reference number indicating the association to which application should be made for nursing help in any particular street. Published at 2s. 6d. (2s. 9d. post free), the Directory can be obtained from the Secretary-Visitor of the Council, 20, Cockspur Street, S.W. 1.

### Lectures on Medical Hydrology.

The University of London Board, in co-operation with the Committee for the Study of Medical Hydrology in Great Britain, has arranged a short course of lectures on Medical Hydrology to be held at the University of London, Imperial Institute, South Kensington. These lectures, which will be supplemented by demonstrations and clinical lectures at Bath, begin on May 6 and end on May 9, the visit to Bath being included in these dates. Application forms with the fee of two and a half guineas must be sent in not later than May 2, and medical men at Bath have kindly agreed to provide hospitality for practitioners and students attending the course. Communications should be sent to the Hon. Secretaries, Committee for Study of Hydrology, care of the University Extension Department of the University of London, South Kensington, S.W. 7.

### College of Nursing Scholarships.

Three scholarships are being offered in June to members of the College of Nursing to enable them to take one year's training at King's College for Women to qualify as sister tutors. Two of the scholarships, valued at £135 each, have been given by Lady Cowdray, and one valued at £150 by Messrs. Boots Pure Drug Company. Applicants will be required to sit for an examination in General and Professional Knowledge on Saturday, June 7. The London Centre of the College of Nursing is offering two Midwifery Scholarships, valued at £12 10s. each, to members of the London Centre wishing to obtain the certificate of the Central Midwives' Board. This examination will also be held on June 7. Forms and information can be obtained from the secretary, and applications to sit for either examination must be made to the Secretary of the College of Nursing, 7 Henrietta Street, W. 1, before May 24.

### Child Welfare Progress in West Riding.

There are now sixty-six maternity and child welfare centres, with a staff of 160 nurses, in the West Riding. The most recent one opened is at the West End Council School, Hemsworth, in an area which has a high infant mortality rate—84 against 69 for the whole country. Dr. Kaye, the Medical Officer for the West Riding, said that the question of child welfare had taken enormous strides during the last two or three years, both in the West Riding and all over the country. It was tragic to enter a school in which approximately 50 per cent. of the children were more or less defective. Many scholars were incapable of taking advantage of the opportunities presented by our excellent system of education. He thought it was lamentable how many young women left school knowing nothing about married life, and he could not understand why members of education committees did not see that personal hygiene occupied a larger share of modern education.

### Hospital Saturday Fund.

A letter from the King was read by Lord Malmesbury (chairman), presiding at the annual general meeting of the Hospital Saturday Fund at the Mansion House on April 12, expressing his satisfaction at the successful work accomplished by the Fund during the past year. That the Fund, in its jubilee year, has been able to record that since its inception very nearly a million and a-quarter pounds have been distributed to London hospitals and allied institutions is, in His Majesty's opinion, a matter of sincere congratulation to all concerned. Presenting the report for 1923, which was adopted, Lord Malmesbury said that £100,765 was received in 1923, which was £3,368 more than in 1922. This was a matter of congratulation to those who had helped as they were working in very difficult and uncertain times and there was competition from other associations engaged on the same work of helping the hospitals. Lord Malmesbury stated further that 69,491 benefits had been granted during the past year by this Fund. Mr. Godfrey Hamilton gave an address on the Voluntary Hospitals system.

### Unveiling of Window at Crumpsall Institution.

On April 3 a stained-glass memorial window was unveiled in the church of the Crumpsall Institution, erected to the memory of those members of the staff who gave their lives during the Great War. The window, which was designed and executed by Mr. J. W. Pearce, of Wilmslow, represents a warrior clad in armour kneeling at the feet of Christ, with arms uplifted, delivering up his sword to his Master, who stoops forward to receive it, while on the right is a shining white-robed angel holding a golden crown.

### King Edward's Hospital Fund.

Lord Somerleyton, secretary of King Edward's Hospital Fund for London, writes:—"It has come to the knowledge of King Edward's Hospital Fund that a circular addressed to 'Employers of Labour, Tradesmen and Philanthropists' has been issued by the 'United Kingdom Free Hospital Insurance Fund,' 17 Ironmonger Lane, E.C. The second paragraph of the circular reads: 'In a speech on the 13th April, 1921, the Prince of Wales expressed a desire that the King Edward's Hospital Fund should be assisted through some means of insurance, and the present scheme has been outlined by the copyright holder of free insurance, the success of which has been phenomenal as evidenced by all the leading newspapers of to-day.' No such statement was made by His Royal Highness. The King's Fund published on April 13, 1921, a report which mentioned insurance as a method that should be thoroughly explored. The King's Fund has since declared itself in favour of the general principle of schemes of mass contributions as suggested by Lord Cave's Committee, and this principle has already been adopted by various hospitals and by the Hospital Saving Association. But the scheme of the United Kingdom Free Hospital Insurance Fund is not one which the King's Fund could approve."

### Chadwick Public Lectures.

Visitors to London during the next two months will have the opportunity of attending a specially interesting series of Chadwick Lectures, which are free. On Friday, May 9, at 5.15 p.m., under the title of "Some Causes of a C3 Population," Professor MacBride, F.R.S., vice-chairman of the Eugenics Education Society, will propound the theory of Mutations, describing some experiments and making important suggestions as to causes of abnormalities which may be operative in producing C3's. The Dean of St. Paul's will take the chair. This lecture will be given in the Hall of the Royal Society of Arts, John Street, Adelphi. Of equal interest, although in a different field of sanitation, will be the history and present position of "Women in Dangerous Trades," by Miss Constance Smith, O.B.E., H.M. Deputy Chief Inspector of Factories, on May 27 at 8 p.m. in the Lecture Hall of Bedford College. The last lecture will be given in the Chelsea Physic Garden, Swan Walk, Chelsea Embankment, on June 4 at 5 p.m. by Sir David Prain, who will speak on "Economic and Hygienic Relationships of Cinchona Bark and Quinine." Further information about Chadwick Lectures may be obtained of the Secretary, Mrs. Aubrey Richardson, O.B.E., at the Offices of the Trust, 13 Great George Street, Westminster.

